# Supplementary material for: Unraveling the Keratin Expression in Oral Leukoplakia: A Scoping Review
Source: Int J Mol Sci. 2024 May 21;25(11):5597. doi: 10.3390/ijms25115597 (PMC11172080; doi:10.3390/ijms25115597)
Supplement: Supplementary file 1 [file ijms-25-05597-s001.zip › ijms-2976182-supplementary.pdf]

Table S1: SEARCH STRATEGIES

PUBMED

Search terms:

#1	"Mouth"[Mesh] OR "Lip"[Mesh] OR "Gingiva"[Mesh] OR "Mouth Floor"[Mesh] OR "Palate, Hard"[Mesh] OR "Palate, Soft"[Mesh] OR "Mouth Mucosa"[Mesh] OR "Tongue"[Mesh]
#2	("oral"[Title/Abstract]) OR ("lip"[Title/Abstract]) OR ("mouth"[Title/Abstract]) OR ("labial"[Title/Abstract]) OR ("gingiva"[Title/Abstract]) OR ("gingival"[Title/Abstract]) OR ("floor of mouth"[Title/Abstract]) OR ("hard palate"[Title/Abstract]) OR ("soft palate"[Title/Abstract]) OR ("alveolar mucosa"[Title/Abstract]) OR ("buccal"[Title/Abstract]) OR ("tongue"[Title/Abstract]) OR ("lingual"[Title/Abstract])
#3	"Keratins"[Mesh]
#4	"cytokeratin"[Title/Abstract]
#5	"Carcinoma in Situ"[Mesh]
#6	"carcinoma in situ"[Title/Abstract]
#7	"Leukoplakia"[Mesh]
#8	"leukoplakia"[Title/Abstract]
#9	"Dysplasia"[Title/Abstract]

Search	Results
#1 AND #3 AND #5	18
#1 AND #3 AND #6	14
#1 AND #3 AND #7	79
#1 AND #3 AND #8	35
#1 AND #3 AND #9	57
#1 AND #4 AND #5	4
#1 AND #4 AND #6	8
#1 AND #4 AND #7	18
#1 AND #4 AND #8	8
#1 AND #4 AND #9	27
#2 AND #3 AND #5	21
#2 AND #3 AND #6	19
#2 AND #3 AND #7	113
#2 AND #3 AND #8	58
#2 AND #3 AND #9	90
#2 AND #4 AND #5	9
#2 AND #4 AND #6	14
#2 AND #4 AND #7	25
#2 AND #4 AND #8	21
#2 AND #4 AND #9	66
Total	704
After removing duplicates	244

SCOPUS

Search strategy:
(cytokeratin) AND (oral) AND (leukoplakia) AND (LIMIT-TO (EXACTKEYWORD , "Cytokeratin") OR LIMIT-TO (EXACTKEYWORD , "Leukoplakia, Oral"))

Total: 144

WEB OF SCIENCE
Search strategy:

((TS=(oral)) AND TS=(leukoplakia)) AND TS=(cytokeratin)

38 results

((ALL=(oral)) AND ALL=(dysplasia)) AND ALL=(cytokeratin)

77 results

Total: 115
After removing duplicates: 97

OVID

Search strategy:
Databases:
- Biosis Previews 1969 to 2024 Week 12
- Biosis Previews 1990 to 2024 Week 12
- AMED (allied and complementary medicine) 1985 to October 2023
- Embase 1974 to 2024 Feb 12
- Embase Classic + Embase 1947 to 2024 February 12
- Ovid MEDLINE® ALL 1946 to February 12 2024

Fields: All fields (af), Key Heading word (kf)
Searches:
- (oral)af AND (cytokeratin)af AND (dysplasia)af
  Limits: article, English language and removal of duplicates
  144 results
- (oral)af AND (cytokeratin)af AND (leukoplakia)af
  Limits: article, English language and removal of duplicates
  77 results
- (oral)af AND (cytokeratin)kf AND (leukoplakia)kf
  4 results
- (oral)af AND (keratin)kf AND (leukoplakia)kf
  9 results
- (oral)af AND (cytokeratin)kf AND (dysplasia)kf
  6 results
- (oral)af AND (keratin)kf AND (dysplasia)kf
  9 results

Total: 249
After removing duplicates: 204
